# Empowered to Stay Active: Psychological Empowerment, Retirement Timing, and Later Life Work

**DOI:** 10.1007/s10804-023-09453-8

**Published:** 2023-05-25

**Authors:** Ivana Drazic, Carsten C. Schermuly, Victoria Büsch

**Affiliations:** Department of Business Psychology, SRH Berlin University of Applied Sciences, Ernst-Reuter-Platz 10, 10587 Berlin, Germany

**Keywords:** Aging workforce, Psychological empowerment, Retirement age, Activeness after retirement, Bridge employment

## Abstract

Motivating older employees both to prolong their working lives and to stay active even after retirement has become increasingly important due to rising old-age dependency ratios. Later life work—including both paid work and volunteering—has thus become an important topic for scholars and practitioners. We aim to extend research on later life work by hypothesizing that psychological empowerment at work increases not only desired and actual retirement ages but also levels of later life work. Second, we test differential effects of psychological empowerment on later life work, expecting it to be more strongly related to paid work after retirement (i.e., bridge employment) than to volunteering. Third, we suggest that the relationship between psychological empowerment and bridge employment depends on the employees’ level of physical limitations. We used data from a longitudinal panel study in Germany in which structured telephone interviews were conducted. A sample of older individuals who had retired between two waves of measurement was drawn (time lag: three years; *n* = 210). The results of a path analysis support the postulated mediation. Furthermore, as expected, psychological empowerment more accurately predicted bridge employment than volunteering, and physical limitations moderated the relationship between psychological empowerment and bridge employment. Lastly, additional analyses on the individual empowerment facets revealed that only the competence facet played a significant role in the proposed hypotheses. Overall, our findings suggest that psychological empowerment may help to increase older employees’ motivation to delay retirement and to stay active even after retirement.

The working-age population in many developed countries is expected to decrease by more than one-third by 2060 (OECD, 2019b). Many aging societies will thus have to face an immense financial burden to their public pension and healthcare systems (Rouzet et al., 2019). Therefore, in countries such as Germany, for example, several economic institutes have recently called for the statutory retirement age to be raised to 69 years (Holtemöller et al., 2021). However, even if such increases would provide a legal basis for longer working lives, the question remains as to whether older employees would actually be able and motivated to delay retirement. Over the years, individual retirement has become a complex and dynamic process (Giandrea et al., 2010). It depends not only on official retirement ages but also on individual contexts, needs, and preferences as well as socio-economic factors (Wang & Shultz, 2010). Involuntary early retirement—that is, “a retirement that results from a situation with (often unexpected) employment constraints” (Dorn & Sousa-Poza, 2010, p. 427)—has increased during the COVID-19 pandemic and is mostly experienced by individuals working in low-wage sectors (Hofäcker & Naumann, 2015; Truesdale, 2020). The main reasons people retire involuntarily are that they work in poor-quality jobs and experience low employment stability during their 50 s (i.e., they are not continuously employed). Thus, it is important to note that, although statutory retirement ages are being raised, many older workers are not able to continue working, at least, because delaying retirement requires having a job to retire from (Truesdale, 2020). At the same time, although early retirement (voluntary or involuntary) is widespread in many developed countries (OECD, 2019a), increasing numbers of older individuals are deciding to prolong their working lives (Grigoli et al., 2021). This heterogeneity raises the following question: Which factors influence the retirement timing decision of older employees who could theoretically continue working?

Common variables that have been linked to retirement intentions and, in consequence, retirement decisions are individual attributes (e.g., health), family factors (e.g., marital status), and socio-economic factors (e.g., current economic conditions; for a review, see Wang & Shultz, 2010). In addition to these factors, job and organizational factors—that is, characteristics of jobs and work organizations and interactions between the person and job variables—also play an important role (see Wang & Shultz, 2010). For example, a study by Vignoli et al. (2021) indicates that higher levels of work ability and lower perceptions of age stereotypes at the workplace are related to the desire to work longer. Another recent study that investigated actual retirement decisions found that individual growth need and organizational climates that encourage older workers’ learning and development were positively related to older workers’ decisions to stay (vs. retire) regardless of their retirement eligibility (Li et al., 2022). Findings from a study by Sousa-Ribeiro et al. (2021) corroborate the importance of an age-friendly work environment and further showed that feeling positive regarding the future at work was positively related to the participants’ preferred, expected and actually retirement ages. In line with this finding, Fasbender et al. (2019) found that career adaptability (i.e., the ability and willingness to manage one’s own career) and levels of personal growth were positively related to late career planning among a sample of older workers, and that both relationships were mediated by occupational future time perspective. Findings from a study by Watermann et al. (2023) underscore the importance of both older individuals’ future time perspectives and perceptions of age discrimination. The authors found that age discrimination was negatively related to retirement intentions via remaining time (a facet of occupational future time perspective). In general, job and organizational factors seem to be particularly relevant because the average employee spends many hours at work before retiring; in Germany, the time spent is approximately 76,500 h (Schermuly, 2016). Thus, organizations may influence older workers’ retirement decisions by changing certain job and organizational factors. Meta-analytical findings suggest that job satisfaction and work involvement are the strongest predictors of retirement planning at the person-job level (see Topa et al., 2009). A more recent systematic review indicates that high job satisfaction and high job control are associated with not only later retirement intentions, but also actual retirement (Browne et al., 2018). However, a meta-analysis by Topa et al. (2018) suggests that the effect of job satisfaction on early retirement decisions is negligible.

A construct at the person-job level that might more consistently predict retirement timing by reaching the root of work-related experiences is *psychological empowerment*. Initially, research on employee empowerment concentrated on socio-structural empowerment (see, e.g., Kanter, 1977), which can be defined as the distribution of power across organizational participants through empowering policies and practices (Liden & Arad, 1996). Although this democratic approach received great attention, it was found to be limited. For example, it could not explain why some employees did not feel empowered even though they were working in empowering structures or vice versa (Spreitzer, 2008). Because of these limitations, researchers started studying employees’ subjective experiences of empowerment. Conger and Kanungo (1988) substantially influenced this change in the view of empowerment (Spreitzer, 2008). Thomas and Velthouse (1990) built on their work by defining empowerment as an intrinsic motivation, further specifying its cognitive preconditions. Finally, based on Thomas’ and Velthouse (1990) work, Spreitzer (1995) proposed a definition of psychological empowerment that is now used widely in empirical research. Spreitzer (1995) defined psychological empowerment as intrinsic task motivation manifested in four work-related cognitions: meaning, self-determination, competence, and impact. *Meaning* reflects the degree of alignment between employees’ values, beliefs, and behaviors and the corresponding requirements of their work roles (Spreitzer, 1995). *Competence* represents the extent to which employees believe that they have the skills to perform their work well (Spreitzer, 2008). *Self-determination* comprises “autonomy in the initiation and continuation of work behaviors and processes” experienced by employees (Spreitzer, 1995, p. 1443). *Impact* reflects how strongly employees can influence important outcomes at work (Spreitzer, 1995). Taken together, these four work-related cognitions constitute the “Gestalt” of psychological empowerment, which implies that all four cognitions are essential for experiencing psychological empowerment (Spreitzer, 1995). Thus, for instance, individuals who perceive a great deal of autonomy but no meaning at work will not feel psychologically empowered (Spreitzer, 2008). In contrast, individuals who do feel psychologically empowered are more likely to take an active orientation toward work (Spreitzer, 1995).

Meta-analytical findings indicate positive consequences of psychological empowerment for important work outcomes such as job satisfaction and performance (Seibert et al., 2011). With increasingly complex and dynamic business environments, research on psychological empowerment—as one answer to this challenge—has blossomed over the past decades (see Seibert et al., 2011), and the topic still generates considerable research interest (e.g., Malik et al., 2021). By enabling employees to use their initiative in their daily work, psychological empowerment can be seen as an important measure to counteract the challenges of increasingly complex business environments (Lee & Edmondson, 2017). However, another major challenge for many companies is the aging of the workforce, and little research on empowerment has focused on the specific empowerment of older employees and its consequences (Naghavi et al., 2019). As one exception, Schermuly et al. (2017) found a positive relationship between psychological empowerment and desired retirement age (DRA). However, their study was based on cross-sectional data, and it did not investigate actual retirement age (ARA). Consistent with this limitation, Browne et al.’s (2018) systematic review calls for more studies to examine ARA when investigating the influence of psychosocial work conditions on retirement decisions. The first part of the present study addresses this research gap by examining whether empowered employees not only desire to retire later but indeed retire later.

The second part of the study investigates the consequences of psychological empowerment on activeness *after* retirement. We hypothesize that psychological empowerment during working life is positively related to levels of later life work. Later life work can be defined as “work activities of older adults just before and beyond normal retirement age” (Wöhrmann, 2021, p. 12), including both paid work and volunteering. Based on continuity theory (Atchley, 1989), we expect psychological empowerment to be related to later life work and to better predict engagement in paid work after retirement than engagement in volunteering. Furthermore, as suggested by Beehr and Bennett (2015), we investigate boundary conditions of the relationship between psychological empowerment and the decision to engage in bridge employment. We suggest that the positive relationship between psychological empowerment and bridge employment is moderated by employees’ physical limitations. Finally, to advance research on psychological empowerment, we respond to calls by Spreitzer (2008) and investigate the roles of the individual facets of psychological empowerment within exploratory analyses.

In summary, we aim to contribute to the literature on later life work by testing whether employees who feel more psychologically empowered (a) not only desire to retire later but indeed retire later, (b) are more likely to engage in paid work after retirement than employees who feel less psychologically empowered, and (c) are more likely to engage in paid work after retirement than in volunteering. Furthermore, we want to shed light on boundary conditions of the proposed relationships by proposing physical limitations as a moderating variable. We also examine the role of the individual facets of psychological empowerment. From a conceptual point of view, these investigations are important, because psychological empowerment reaches the root of work-related experiences by reflecting how people interpret their job situation. It precedes common person-job factors such as job satisfaction and represents a more complex antecedent of later life work, comprising four distinct facets. Finally, from a practical point of view, it is worthwhile to study psychological empowerment, as research suggests that it is malleable. Meta-analytical findings indicate that it can be influenced by psychosocial organizational factors such as structural empowerment, leadership, and trust in the organization (Llorente-Alonso et al., 2023; Seibert et al., 2011). To test our hypotheses, we extended previous research on psychological empowerment and DRA using panel data from the project “Transition and Old Age Potential” (TOP). Individuals aged 55 years or over were interviewed via telephone. We applied a two-wave design with a time lag of three years, investigating individuals who had retired between two waves of measurement (*n* = 210). In the next paragraph, we first provide information on the German retirement system to contextualize our study. Second, we conceptualize retirement from a psychological perspective and introduce the temporal model of retirement as an overarching framework. Lastly, we derive our hypotheses. The conceptual research model for all hypotheses is depicted in Fig. 1.

**Fig. 1:**
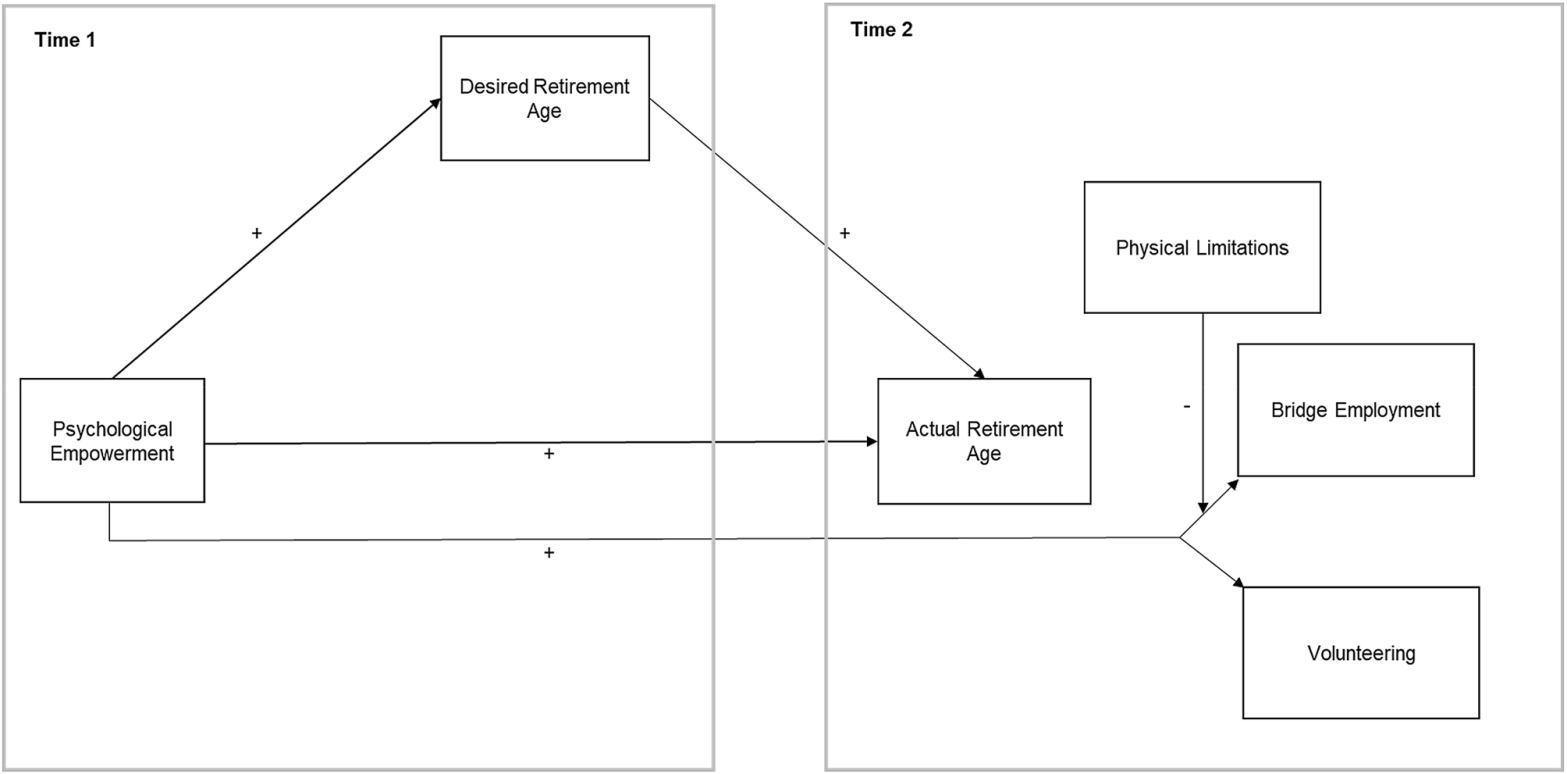
Conceptual research model

## Background

### The German Retirement System

Germany has a long history of institutionalized early retirement (i.e., the act of retiring before the statutory retirement age), which was intended, inter-alia, to promote organizational restructuring (Trampusch, 2005). Not until the 1990s did German policymakers gradually begin to close pathways to early retirement (Hofäcker & Naumann, 2015). In the 2000s, Germany further promoted a paradigm shift from early exit to working longer by introducing labor market programs for older employees and a stepwise increase of the statutory retirement age from 65 to 67 years between 2011 and 2029 (BMAS, 2020; Hofäcker & Naumann, 2015).

In general, the current German pension program allows for flexible retirement timing: Individuals can retire before reaching statutory retirement age by accepting monthly pension claims, which are to be reduced by 0.3% until statutory retirement age is reached. On the other hand, surpassing statutory retirement age is also possible and even gets rewarded with a surcharge of 0.5% per month (Czepek & Weber, 2015). Furthermore, retirees who do not retire early can continue working without income restrictions (DRV, 2020), which further establishes financial incentives to prolong working life. Concerning the sample of the present study, the statutory retirement ages for claiming full state pension ranged from 65 to 66 years.

### The Temporal Process Model of Retirement

Retirement has been conceptualized in many different ways in the literature, with the lowest common denominator being that it is not a single event, but rather a process that takes place over a period of time (Shultz & Wang, 2011). For a long time, the focus in retirement research has been on physical and financial factors of retirement (Shultz & Wang, 2011). However, over the last decades, an increasing amount of research has applied psychological perspectives on the retirement process. Wang and Shi (2014) identified three psychological conceptualizations of retirement based on their literature review: (1) retirement as decision making, (2) retirement as an adjustment process, and (3) retirement as a career-development stage. In the conceptualization of retirement as decision making, older workers consider and weigh information that they have about themselves, their work, and their private life to make decisions about their retirement. Retirement as an adjustment process focuses on the transition from employment to retirement and the individual development of older peoples’ lives after retirement. Finally, retirement as a career-development stage stresses older people’s ongoing potential to change their own career paths after retirement (Wang & Shi, 2014). These conceptualizations are not completely separate from each other but can be viewed as parts of an overarching process (see, e.g., Wöhrmann, 2021).

One framework that helps to integrate the different conceptualizations of retirement is the temporal process model of retirement (e.g., Wang & Shi, 2014; Wang & Shultz, 2010). This model describes the retirement process heuristically, capturing its dynamic and complex nature. It outlines three sequential and gradually unfolding retirement stages: (1) retirement planning, (2) retirement decision making, and (3) retirement transition and adjustment. In the retirement planning phase, older people begin to envision their still distant retirement lives and make initial plans and preparations for retirement. As retirement nears, people enter the decision-making phase. In analogy to the conceptualization of retirement as decision making, in this phase, individuals consider and weigh concrete alternatives to reach conclusions about their retirement. The final phase, retirement transition and adjustment, deals with daily changes in retirement life after the transition from full-time worker to retiree. Daily activities in retirement include leisure activities, caregiving, and later life work (i.e., paid and/or unpaid forms of work). As well as describing the sequential process of retirement, the temporal process model identifies four factors influencing the retirement process that we have already mentioned in the Introduction: individual attributes, family factors, socio-economic factors, and job and organizational factors.

The first part of the present study examines the relationship between psychological empowerment and retirement timing (i.e., desired and actual retirement ages) and thus focuses on the first two phases of retirement: retirement planning and retirement decision making. The second part of the study examines the relationship between psychological empowerment during working life and later life work (i.e., bridge employment and volunteering). Thus, this part of the study focuses on the third phase of the retirement process: retirement transition and adjustment. Following suggestions about the correspondence between certain retirement conceptualizations and certain theories (see Wang & Shultz, 2010), we conceptualize retirement as an adjustment and a decision-making process and focus on continuity theory (Atchley, 1989) and the theory of planned behavior (Ajzen, 1991), respectively.

#### Retirement Planning and Decision Making: The Relationship between Psychological Empowerment, Desired Retirement Age, and Actual Retirement Age

As suggested by the temporal process model of retirement (e.g., Wang & Shultz, 2010), retirement typically starts with a phase in which older people gather and weigh information about themselves, their work, and their private life to define when and how to retire. We argue that older individuals use their psychological empowerment experiences as a source of information from the job and work field and that empowered older individuals want to retire later than less empowered individuals. We base this hypothesis on continuity theory (Atchley, 1989), which is in line with the conceptualization of retirement as an adjustment process. Continuity theory focuses on adaptation to change in later life stages, and it states that people strive to maintain internal continuity (i.e., their self-concept) and external continuity (i.e., social structures) across their lifespan to prevent experiences of harmful disruption. A central premise of continuity theory is that individuals maintain continuity by engaging in types of activities, behaviors, and relationships similar to those they have previously experienced. We argue that the more psychological empowerment older individuals experience in their preretirement lives, the more they will wish to continue this experience in order to maintain continuity. We hypothesize that this desire for continuity will be reflected in the desire to delay retirement, as remaining in the workforce allows them to continue to experience psychological empowerment. Previous research has corroborated this assumption. Schermuly et al. (2017), for example, found that the more psychologically empowered employees felt, the later they wanted to retire.

With the present study, we aim to extend previous findings on the relationship between psychological empowerment and the delay of retirement by investigating whether psychologically empowered employees not only desire to retire later but actually retire later. As stated above, retirement can be conceptualized as a motivational decision-making process (Shultz & Wang, 2011). In motivational processes, intentions are the most proximal determinants of actual behavior (see theory of planned behavior; Ajzen, 1991). According to the theory of planned behavior, an intention reflects the extent to which an individual is motivated to perform a specific behavior, and it is driven by attitudes toward the behavior, subjective norms, and perceived behavioral control. First, we believe desires constitute a valid proxy for intentions, as desires have been shown to mediate the relationship between attitudes, norms and perceived behavioral control, and intentions (see Perugini & Bagozzi, 2001). Second, we argue that in the case of retirement, perceived behavioral control plays a particularly important role: Retirement timing is, to a great degree, dependent on legal regulations. Thus, in countries where pension programs allow for flexible retirement timing—such as Germany—the desire to retire should have a major influence on ARA. Based on these arguments, we hypothesize:

##### *Hypothesis 1*

Higher levels of psychological empowerment lead to higher levels of DRA, which in turn result in higher levels of ARA.

#### Retirement Transition and Adjustment: The Relationship between Psychological Empowerment and Work after Retirement

Even for individuals with high levels of psychological empowerment, there comes a time when it is reasonable to retire—for example, for financial reasons. However, even after retirement, older people can continue to experience psychological empowerment by engaging in work after retirement. Work after retirement can be paid or nonpaid (see definition of later life work; Wöhrmann, 2021). We believe that psychological empowerment during working life predicts levels of later life work, especially paid work after retirement, which is often discussed as *bridge employment* in the literature (Shultz, 2003).

Bridge employment refers to the pattern of labor force participation of older employees that spans the period between their career jobs and complete withdrawal from the labor force (Shultz, 2003; Wang et al., 2008). A simpler definition of bridge employment is any kind of paid work after retirement (e.g., Shultz, 2003). It can be part-time, full-time, or self-employed, career- or noncareer-related, with the same or a different employer (for a taxonomy, see Beehr & Bennett, 2015), and it can be dynamic (individuals can enter and leave the workforce several times after retirement; Wang et al., 2009). The most typical categorization of bridge employment is that of career bridge employment (i.e., employment in the same industry or field as the career job) in contrast to bridge employment in a different field (Shultz, 2003; Wang et al., 2008).

Antecedents of bridge employment can be categorized into personal characteristics (e.g., health), contextual factors (e.g., economy), and organizational factors (e.g., policy incentives; see Beehr & Benett, 2015). It is assumed that organizational factors are indirectly related to bridge employment, mediated by subjective attitudes and feelings toward the organization. Indeed, cognitions relating to work and the organization have been linked to bridge employment. For example, a study by Garcia et al. (2021) found that organizational support increased bridge employment intentions only among older workers who endorsed a relational psychological contract (vs. a transactional psychological contract). Other recent studies suggest that experiences of work meaningfulness mediate the relationships between workplace age discrimination, workplace incivility, relational job crafting (i.e., interpersonal crafting at work), and generativity opportunities and bridge employment intentions, respectively (Peng, 2022; Peng et al., 2020). Another study focusing on employee’ cognitions found that employees with higher outcome expectations regarding post-retirement work (e.g., they had greater expectations that post-retirement work would enable them to stay physically and mentally healthy) were more likely to engage in post-retirement work planning (Wöhrmann et al., 2013). These findings substantiate the assumption that it is not the organizational factors per se but the subjective interpretations of workers that determine how likely they are to engage in bridge employment.

We hypothesize that psychological empowerment plays such an important mediating role between organizational factors and the bridge employment decision making by capturing employees’ subjective experiences of their work. Compared to cognitions of outcome expectations (see Wöhrmann et al., 2013) or experienced meaningfulness of work (Peng, 2022; Peng et al., 2020), psychological empowerment is a broader construct focusing on the present state of employees’ interpretations of their work and therefore may represent a more immediate predictor of bridge employment. Furthermore, in contrast to more general job-related constructs that have been identified as antecedents of bridge employment, such as job satisfaction, commitment, intrinsic motivation, or work stress (see, e.g., Beehr & Bennett, 2015; Wang et al., 2008), psychological empowerment allows for more fine-grained insights, as it consists of four facets that might serve as potential leverages to promote bridge employment.

From a theoretical point of view, we draw from continuity theory (Atchley, 1989), which we have introduced above. Atchley (1989) argues that “adults employ concepts of their past to conceive of their future and structure their choices in response to the changes brought about by normal aging” (p. 183). Retirement constitutes such a normal change. This striving for continuity in one’s self-concept leads older workers to envision themselves being and acting rather similarly in retirement as in the present moment (Feldman, 2013). Thus, following continuity theory (Atchley, 1989), we suggest that older workers who have experienced psychological empowerment during working life will choose circumstances after retirement that provide them with similar experiences. We argue that bridge employment constitutes the most proximal setting in which retired individuals can continue to experience psychological empowerment.

##### *Hypothesis 2a*

Psychological empowerment before retirement is positively related to engagement in bridge employment.

To further corroborate continuity theory (Atchley, 1989), we hypothesize that psychological empowerment before retirement is more strongly related to bridge employment than to volunteering after retirement. Although a recent study has shown that psychological empowerment can be stimulated in the volunteering context (Traeger & Alfes, 2019), we believe the context of the original empowerment experience to be crucial when it comes to continuity. In line with this argument, a recent longitudinal study by Vangen et al. (2021) found that previous engagement in voluntary work among 62- to 75-year-olds in Norway was strongly associated with voluntary work 10 years later. We agree with Vangen et al. (2021), who state that “being active in older age may depend on earlier activity patterns” (p. 2). Thus, we believe that, if psychological empowerment has been experienced in the work context, it is more likely that individuals will seek opportunities in this life arena to continue experiencing empowerment.

##### *Hypothesis 2b*

Psychological empowerment before retirement is more strongly related to bridge employment than to volunteering after retirement.

Finally, drawing from the model of the bridge employment decision-making process by Beehr and Bennett (2015), we suggest that the relationship between psychological empowerment and bridge employment is influenced by boundary conditions. Beehr and Bennett (2015) built on the process model of retirement (Wang & Shultz, 2010), adding situational constraints (e.g., unexpected factors such as additional expenses or health problems) as boundary conditions to the model. We assume that physical limitations are an important boundary condition, as poor health is one of the most important reasons for an early exit from the labor force (see, e.g., van Rijn et al., 2014; Vanajan et al., 2020) and has been shown to interact with worker characteristics such as age and race (see a review by Fisher et al., 2016). High physical limitations reflect the individual’s inability to perform daily activities such as sitting for more than two hours or lifting something, implying a “limited capacity to meet the requirements of core social, familial, and occupational roles” (Gayman et al., 2008, p. 219). From a theoretical perspective, continuity theory (Atchley, 1989) might explain the potential boundary effect of physical limitations on bridge employment decision making. Continuity theory (Atchley, 1989) states that “external continuity is usually an effective adaptive strategy but can sometimes be maladaptive” (p. 189). According to Atchley (1989), external continuity (e.g., continuing to work after retirement) becomes maladaptive when the physical and mental abilities required for continuity are seriously impaired. We hypothesize that the relationship between psychological empowerment and bridge employment depends on the level of physical limitations, such that the relationship between psychological empowerment and bridge employment can only unfold when physical limitations are low. Consequently, we argue that older people are more likely to use adaptive strategies—that is, to retire when sufficient physical resources for continuing to work are no longer available—than to force external continuity, even though they might report high levels of psychological empowerment.

##### *Hypothesis 2c*

The positive relationship between psychological empowerment and bridge employment is moderated by physical limitations, such that the relationship is stronger for retirees with low levels of physical limitations and weaker for retirees with high levels of physical limitations.

## Method

### Participants and Procedure

We used data from the TOP study, a longitudinal, population-representative panel study, carried out on behalf of the German Federal Institute for Population Research (Mergenthaler et al., 2017). It investigated older adults’ potential in Germany and their transition from working life to retirement. Standardized and computer-assisted telephone interviews were conducted by trained interviewers. To ensure random sampling of households with a landline number, the Gabler-Häder design was used, which considers listed and unlisted numbers equally (Sackreuther et al., 2016). For our research question, we used data from the first wave (T1; January–April 2013) and the second wave (T2; November 2015–February 2016). At T1, a total of 5,002 respondents aged 55–70 years completed the interview, 50% of whom also completed the interview at T2 (Mergenthaler et al., 2017). Given our research question, we investigated only respondents who had retired between T1 and T2; thus, individuals who (a) were working part- or full-time at T1 and were not yet retired (*n* = 997) and (b) were retired at T2 (*n* = 222). We excluded 12 participants who had missing values on the empowerment items, showed logical inconsistencies regarding their retirement year, or did not indicate their retirement year at all. This resulted in a final sample of 210 participants. Furthermore, we replaced the DRA of eight participants with missing values because of extreme data (i.e., 2 *SD* below or above the mean). Missing values were generally accounted for by full-information maximum likelihood (FIML) estimation or listwise deletion, depending on the model used (see section on statistical analyses below).

Participants at T1 were between 54 and 65 years old (*M* = 61.97, *SD* = 1.95); 50% were female, and 77.1% indicated being in a committed relationship. According to the International Standard Classification of Education 97 classification, most participants (58.6%) had a high level of education (up to a doctoral degree or equivalent). At T1, participants worked 37.3 h per week on average (*SD* = 12.2). Ten percent were blue-collar workers, 51.4% white-collar workers, 23.3% civil servants, 13.3% self-employed, and 1.4% supported their family business. Most participants worked either in small companies (less than 50 employees; 36.7%) or large companies (more than 250 employees; 37.1%). Table 1 summarizes the sample characteristics measured at T1.

**Table 1:** Sample characteristics at T1

	n/M	%/SD
*Gender*		
Female	105	50
Male	105	50
*Age*	*M* = 61.97	*SD* = 1.95
*Working hours*^a^	*M* = 37.29	*SD* = 12.20
*Relationship status*		
Committed relationship	162	77.14
No committed relationship	48	22.86
*Education (ISCED)*		
Low	4	1.91
Middle	83	39.53
High	123	58.57
*Position*^b^		
Blue-collar	21	10.00
White-collar	108	51.43
Civil servants	49	23.33
Self-employed	28	13.33
Support in family business	3	1.43
*Organizational size*^c^		
Small (less than 50 employees)	77	36.67
Medium-sized (between 50 and 250 employees)	37	17.62
Large (more than 250 employees)	78	37.14
*Household net income (in euros)*^d^	*M* = 2574.82	*SD* = 2970.37

*n* = 210;

^a^*n* = 203; ^b^*n* = 209; ^c^*n* = 192; ^d^*n* = 207

### Measures

The interview guide for the TOP study was developed and pre-tested by the German Federal Institute for Population Research in cooperation with several universities. Unless otherwise indicated, we recoded all items so that higher values indicated higher scale expressions.

#### ARA (T2)

ARA was measured with a single item asking participants to indicate the year they retired. We then subtracted the participants’ year of birth from their retirement year.

#### DRA (T1)

Participants were asked to indicate the year they would like to retire. Again, we calculated the difference scores using the participants’ year of birth.

#### Bridge Employment (T2)

Participants were asked whether they were currently engaged in paid work (they were prompted to think of any kind of paid work after retirement). Of the 210 participants, 78 (37.1%) indicated that they were engaged in bridge employment; one participant did not provide the information.

#### Volunteering (T2)

Volunteering was measured with the question “Have you engaged in any volunteer or honorary activity in the last three months, e.g., participating in an association, initiative, or group?” Of the 210 participants, 100 (47.6%) reported volunteering.

#### Psychological Empowerment (T1)

Psychological empowerment was measured with the same items that were used by Schermuly et al. (2017). The TOP research group adapted the items from Spreitzer’s (1995) original 12-item scale. Especially in the telephone interview context, scales that are designed to be self-administered have been deemed too long and complex (Hughes et al., 2004). Thus, the TOP research group decided to use a selection of Spreitzer’s (1995) empowerment scale and to omit the impact facet altogether, as prior research suggests a close relationship with the self-determination facet (e.g., Kraimer et al., 1999; Schermuly et al., 2013). Meaning and self-determination were measured with single items: “My work is very important to me” and “During work, I have the opportunity to make decisions autonomously,” respectively. Competence was measured with two items: “I face work problems with calmness because I can always rely on my competencies” and “Whatever happens in my job, I will master it.” Participants assessed the items on a 4-point Likert scale ranging from 1 (strongly agree) to 4 (strongly disagree). Concerning reliability, it is increasingly recommended to replace Cronbach’s *α* with alternative coefficients. This is because of its unrealistic assumptions, which oftentimes lead to underestimated reliability scores (McNeish, 2018). Omega coefficients, for example, are more appropriate measures of reliability in case not all items are equally strongly associated with the underlying construct (Peters, 2014). This lack of homogeneity becomes even more likely with shorter scales (Berger, 2019). Revelle’s omega (Revelle & Zinbarg, 2009) for the three-faceted empowerment construct in the present study was 0.61. Although this value is not optimal, it is not surprising: Even in noninterview contexts, lower reliability scores for the empowerment construct are not unusual and deemed acceptable because of the construct’s first-order multidimensionality (Spreitzer, 1995). Schermuly et al. (2017), who used the same items but with a larger sample of the TOP study, conducted exploratory and confirmatory factor analyses, both of which suggested a single empowerment factor after inspecting the scree plot, Eigenvalues, and fit indices. Based on these arguments, we decided to use this interview-adapted empowerment scale and its mean score for subsequent statistical analyses.

#### Physical Limitations (T2)

The level of physical limitations was measured with the question “During the last four weeks, how often were you limited in your activities because of physical problems?” Participants assessed this item on a 4-point Likert scale ranging from 1 (very often) to 4 (never). We also recoded this item so that higher values reflected greater frequency.

### Controls

#### Gender (T1)

In line with other research on bridge employment and retirement (see reviews by Beehr & Bennett, 2015 and Fisher et al., 2016), we controlled for gender.

#### Internal Locus of Control (T1)

Meta-analytical findings suggest a strong relationship between internal locus of control (a core self-evaluation trait) and psychological empowerment (Seibert et al., 2011). Core self-evaluation traits in turn have been found to be positively related to retirement variables such as retirement preparation (Zaniboni et al., 2021). On a 4-point Likert scale ranging from 1 (strongly agree) to 4 (strongly disagree), participants were asked to assess the following two statements: “I am in control of my life” and “If I make an effort, I will succeed.”

#### Public Sector Employment (T1)

We controlled for employees in the public sector, as they have other retirement conditions as private employees in Germany. For example, the German pension law imposes income restrictions on employees from the public sector who have retired (BMAS, 2019). This might alter incentives for bridge employment. Participants in the present study who indicated being civil servants were coded as 1, whereas all other forms of employment were coded as 0.

#### Equivalized Net Income of Households (T1)

We see pay as a resource provided by the organization, and access to resources has been shown to be positively associated with perceptions of psychological empowerment (Seibert et al., 2011). On the other hand, it is well known that a person’s financial situation plays an important role in retirement decisions (see Fisher et al., 2016). We used the modified Organisation for Economic Co-operation and Development (OECD) equivalence scale, which adjusts disposable monthly household income by the number and age of household members (Sackreuther et al., 2016). The median OECD equivalized monthly net income of households was EUR 2076.5.

#### Temporal Latency (T2)

Whether or not someone engages in occupation-related activities after retirement may depend on how long ago one has retired (Fasbender et al., 2016). To calculate temporal latency, we subtracted the year of retirement from the second measurement year. On average, temporal latency was 1.75 years (*SD* = 0.82).

### Statistical Analyses

All hypotheses were tested with the statistical software R Studio. For Hypothesis 1 (mediation analysis), we conducted a path analysis using the maximum likelihood estimator with the R package lavaan (Rosseel, 2012). We further specified 5,000 bootstrap samples, a 95% confidence interval (CI), and FIML estimation for missing values. According to Hooper et al. (2008), the model testing Hypothesis 1 showed good fit indices, *χ*^2^(4) = 5.571, *p* = 0.234, CFI = 0.999, TLI = 0.998, RMSEA = 0.044, SRMR = 0.032. To test Hypotheses 2a and 2c, we conducted a hierarchical binary logistic regression, first entering the control variables (Model 1), then entering the main variable (Model 2), and lastly, entering the moderating variable (i.e., physical limitations). For Hypothesis 2b, we conducted a path analysis using the diagonally weighted least squares (DWLS) estimator to enable the simultaneous inclusion of both categorical dependent variables (i.e., bridge employment and volunteering; see Rosseel, 2014). This model was just identified; thus, examining model fit indices to assess model fit is not meaningful. The control variables gender, locus of control, public sector employee, and equivalized net income of households were included as predictors of ARA; in bridge employment and volunteering, we further controlled for temporal latency.

## Results

### Preliminary Analysis

Table 2 shows descriptive results and intercorrelations. On average, participants wanted to retire at 64.12 years of age (*SD* = 2.18) and retired at 63.93 years (*SD* = 1.83). Psychological empowerment was positively correlated with all dependent variables except for bridge employment and volunteering. Furthermore, psychological empowerment was positively correlated with locus of control, gender was positively correlated with ARA, and volunteering was positively correlated with public sector employment.

**Table 2:** Means (M), standard deviations (SD), and intercorrelations among study variables

Variable (unit/scale)	*M*	*SD*	1	2	3	4	5	6	7	8	9	10
1. ARA (years)	63.93	1.83										
2. DRA^a^ (years)	64.12	2.18	0.67**									
3. Bridge employment^b^ (0 = no, 1 = yes)	0.37	0.48	0.15*	0.20**								
4. Volunteering (0 = no, 1 = yes)	0.48	0.50	− 0.03	− 0.07	− 0.12							
5. Psychol. empowerment (scale 1–4)	3.56	0.40	0.23**	0.26**	0.12	-0.06						
6. Physical limitations (scale 1–4)	1.68	0.86	− 0.21**	− 0.18*	− 0.17*	− 0.16*	− 0.01					
7. Gender (1 = female, 2 = male)	1.50	0.50	0.14*	0.11	− 0.10	− 0.06	0.11	0.02				
8. Locus of control (scale 1–4)	3.31	0.56	− 0.03	− 0.04	0.02	− 0.01	0.16*	− 0.00	− 0.06			
9. Public sector ^b^ (0 = no, 1 = yes)	0.23	0.42	− 0.09	− 0.02	− 0.13	0.24**	− 0.03	− 0.10	0.03	− 0.03		
10. Equiv. net income ^c^ (in EUR)	2574.82	2970.37	0.06	0.12	0.00	0.08	0.11	− 0.12	− 0.03	0.00	0.07	
11. Latency (years)	1.75	0.82	− 0.11	0.11	0.08	0.07	0.05	0.06	− 0.07	− 0.07	− 0.01	0.14*

*p < 0.05. **p < 0.01

*n* = 210; ^a^*n* = 194; ^b^*n* = 209; ^c^*n* = 207

*ARA* actual retirement Age, *DRA* desired retirement age

### Hypothesis Testing

Hypothesis 1 states that higher levels of psychological empowerment lead to higher levels of DRA, which in turn result in higher levels of ARA. The observed indirect effect supports this hypothesis (unstandardized indirect effect: 0.82, 95% CI [0.37; 1.24], *p* < 0.001). Psychological empowerment significantly predicted DRA (*β* = 0.27, 95% CI [0.73; 2.20], *p* < 0.001), which in turn predicted ARA (*β* = 0.65, 95% CI [0.44; 0.68], *p* < 0.001). The direct effect of psychological empowerment on ARA was not significant (*β* = 0.07, 95% CI [− 0.27, 0.87], *p* = 0.288), which indicates full mediation. None of the control variables significantly predicted the ARA (gender, *β* = 0.07, 95% CI [− 0.11, 0.64], *p* = 0.174; locus of control, *β* = − 0.02, 95% CI [− 0.41, 0.29], *p* = 0.762; public sector employment, *β* = − 0.07, 95% CI [− 0.75, 0.22], *p* = 0.246; equivalized net income of households, *β* = − 0.02, 95% CI [− 0.00, 0.00], *p* = 0.838). The model accounts for 7.4% of the variance of DRA and 46.4% of the variance of ARA. Following recommendations by Becker et al. (2015), we also ran the mediation analysis without control variables, which did not change the effects.

In Hypothesis 2a, we assumed psychological empowerment at T1 to be positively associated with bridge employment at T2. The results of the binary logistic regression are presented in Table 3. The control variables entered in step 1 did not predict engagement in bridge employment. Psychological empowerment entered in step 2 significantly predicted bridge employment, indicating that individuals with higher levels of psychological empowerment were more likely to engage in bridge employment. Model 2 accounts for 4% of the variance in bridge employment.

**Table 3:** Results of the binary logistic regression predicting bridge employment

Variable	Model 1					Model 2					Model 3				
	*B*	*SE B*	*p*	95% CI	OR	*B*	*SE B*	*p*	95% CI	OR	*B*	*SE B*	*p*	95% CI	OR
Intercept	− 0.71	1.00	0.478	[− 2.71, 1.24]		− 3.23*	1.64	0.049	[− 6.53, − 0.09]		− 0.19	0.23	0.398	[− 0.64, 0.25]	
Step 1: Control variables															
Gender (1 = female, 2 = male)	− 0.38	0.30	0.198	[− 0.96, 0.20]	0.68	− 0.45	0.30	0.136	[− 1.04, 0.14]	0.64	− 0.50	0.31	0.108	[− 1.12, 0.11]	0.60
Locus of control	0.06	0.26	0.831	[− 0.46, 0.58]	1.06	− 0.04	0.27	0.885	[− 0.57, 0.49]	0.96	− 0.10	0.28	0.736	[− 0.65, 0.47]	0.91
Public sector	− 0.62	0.37	0.092	[− 1.36, 0.08]	0.54	− 0.59	0.37	0.109	[− 1.34, 0.11]	0.55	− 0.70	0.38	0.066	[− 1.48, 0.03]	0.50
Net income	− 0.00	0.00	0.957	[− 0.00, 0.00]	1.00	− 0.00	0.00	0.772	[− 0.00, 0.00]	1.00	− 0.00	0.00	0.464	[− 0.00, 0.00]	1.00
Latency	0.18	0.18	0.315	[− 0.17, 0.54]	1.20	0.18	0.18	0.332	[− 0.18, 0.54]	1.20	0.25	0.19	0.201	[− 0.13, 0.62]	1.28
Step 2: Main variable															
Psychological empowerment						0.81*	0.41	0.049	[0.03, 1.64]	2.24	0.77	0.43	0.072	[− 0.05, 1.63]	2.15
Step 3: Interaction															
Physical limitations											− 0.52*	0.20	0.012	[− 0.93, − 0.14]	0.60
Psychological empowerment × Physical limitations											− 0.96*	0.48	0.045	[− 1.92, − 0.01]	0.38
Model Fit:															
− 2 log likelihood	264.156					260.055					247.578				
Δ − 2 log likelihood (Δdf)						4.101*(1)					12.477**(2)				
Pseudo R2	0.02					0.04					0.08				

In Model 3, all continuous predictors were mean-centered

*n* = 205

*SE* Standard error, *CI* confidence interval, *OR* odds ratio

*p < 0.05, **p < 0.01

**Fig. 2:**
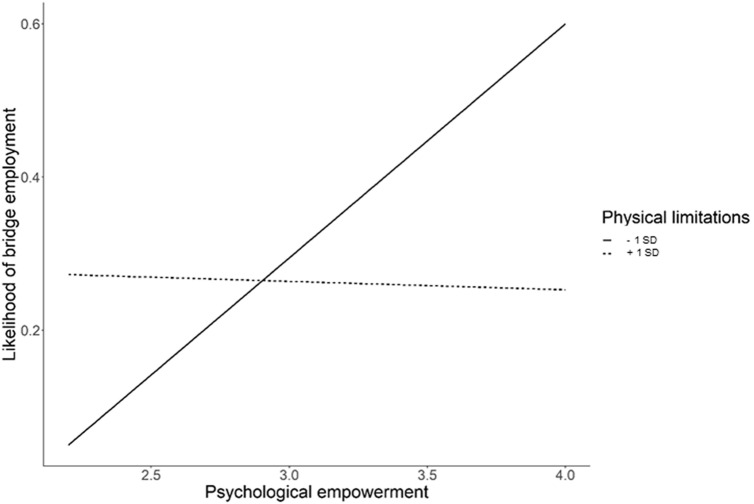
Moderation of the relationship between psychological empowerment and bridge employment by physical limitations

In Hypothesis 2c, we assumed that employees’ physical limitations would moderate the relationship between psychological empowerment and bridge employment. Our data support this hypothesis (see Model 3 in Table 3). The interaction between psychological empowerment and physical limitations was significantly related to the likelihood of engaging in bridge employment. Simple slope analyses revealed that the relationship between psychological empowerment and bridge employment became stronger when levels of physical limitations were low (i.e., 1 *SD* below the mean; *B* = 1.42, *p* = 0.008, CI [0.36, 2.47]). When physical limitations were high (i.e., 1 *SD* above the mean), the relationship between psychological empowerment and bridge employment was nonsignificant (*B* = − 0.07, *p* = 0.910, CI [− 1.22, 1.09]). Figure 2 shows the interaction between psychological empowerment and physical limitations.

In Hypothesis 2b, we assumed psychological empowerment at T1 to be more strongly related to bridge employment than volunteering at T2. The path analysis supports this hypothesis: Psychological empowerment was significant in predicting bridge employment, indicating that individuals with higher levels of psychological empowerment were more likely to engage in bridge employment. In contrast, psychological empowerment did not significantly predict volunteering. Concerning the set of control variables, none of the variables significantly predicted bridge employment, but volunteering was significantly predicted by public sector employment. The model accounts for 9% of the variance in bridge employment and 12% of the variance in volunteering. The results of the path analysis are summarized in Table 4. We also ran this model without control variables, which slightly decreased the statistical significance of psychological empowerment, such that it only significantly predicted bridge employment based on a 90% CI [0.05, 0.80].

**Table 4:** Results of the path analysis predicting bridge employment and volunteering

Variable	Bridge employment					Volunteering				
	*B*	*SE B*	β	*p*	95% CI	*B*	*SE B*	β	*p*	95% CI
Control variables										
Gender	− 0.27	0.19	− 0.13	0.156	[− 0.68, 0.06]	− 0.15	0.19	− 0.07	0.437	[− 0.53, 0.21]
Locus of control	− 0.03	0.18	− 0.02	0.877	[− 0.35, 0.36]	0.02	0.17	0.01	0.917	[− 0.33, 0.32]
Public sector	− 0.36	0.24	− 0.15	0.132	[− 0.90, 0.03]	0.72**	0.23	0.29**	0.002	[0.31, 1.24]
Net income	− 0.00	0.00	− 0.03	0.915	[− 0.00, 0.00]	0.00	0.00	0.10	0.547	[− 0.00, 0.00]
Latency	0.11	0.12	0.09	0.350	[− 0.12, 0.35]	0.10	0.12	0.08	0.400	[− 0.13, 0.33]
Main variable										
Psychological empowerment	0.51*	0.25	0.19*	0.040	[0.04, 1.00]	− 0.19	0.25	− 0.07	0.444	[− 0.67, 0.31]

*N* = 205. For gender, 1 = female, 2 = male

*p < 0.05, **p < 0.01

*CI* confidence interval

### Additional Analyses

**Fig. 3:**
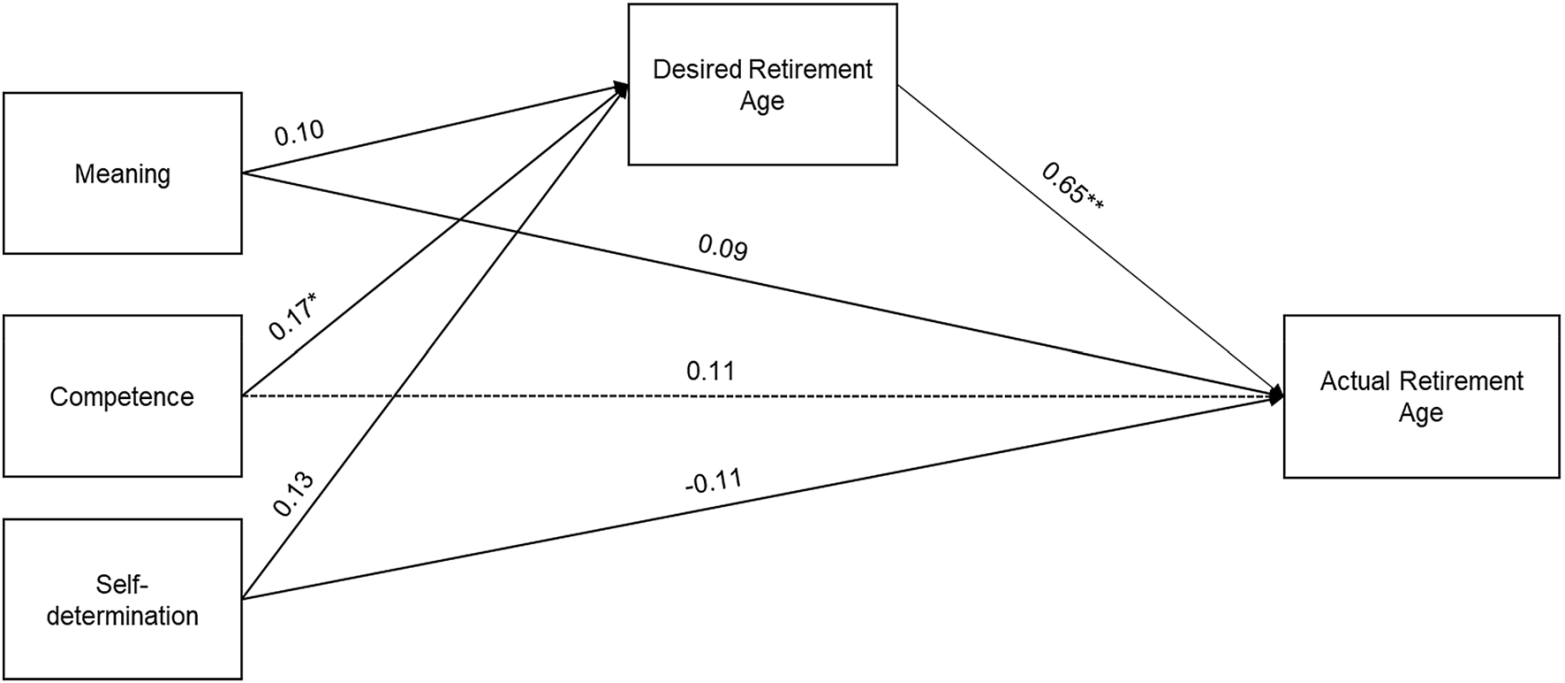
Results of the Mediation Hypothesis with the Individual Facets of Psychological Empowerment as Predictors. *Note*. *p < 0.05. **p < 0.01. All displayed coefficients are standardized. Control variables are omitted in the figure. Dashed line indicates full mediation

To obtain more fine-grained insights, we reran our analyses using the three dimensions of psychological empowerment as individual predictors. For that, we calculated a mean score of the two items measuring the competence facet (self-determination and meaning were measured with single items). Regarding Hypothesis 1, we found that only the competence facet significantly predicted the DRA, which in turn predicted the ARA. The observed indirect effect was significant (unstandardized indirect effect: 0.22, 95% CI [0.04; 0.42], *p* = 0.025), while the direct effect was nonsignificant, indicating full mediation. None of the control variables (i.e., gender, locus of control, public sector employee, or equivalized net income of households) significantly predicted the ARA. Figure 3 shows the model with standardized parameter estimates.

Regarding Hypothesis 2, the results showed that none of the single facets of psychological empowerment significantly predicted bridge employment or volunteering. As in the model with psychological empowerment as a unitary construct, none of the control variables significantly predicted bridge employment, and only public sector employment significantly predicted volunteering (*β* = 0.30, 95% CI [0.29; 1.22], *p* = 0.002). All additional analyses are available upon request from the first author.

## Discussion

Especially in aging societies, successfully motivating older employees to extend their working lives is essential. Using a sample of a longitudinal panel study in Germany, we found that psychologically empowered employees not only desired to retire later but indeed retired later (Hypothesis 1). Second, we tested whether psychological empowerment predicts engagement in bridge employment (Hypothesis 2a). Our findings also support this hypothesis. Furthermore, we hypothesized that psychological empowerment better predicts bridge employment than volunteering (Hypothesis 2b). Psychological empowerment was indeed significant in predicting bridge employment while showing a nonsignificant relationship to volunteering; however, effect sizes were small. Finally, we found that physical limitations moderated the relationship between psychological empowerment and bridge employment, such that the relationship was stronger when physical limitations were low and nonexistent when physical limitations were high. In the next section, we will interpret these findings in light of existing theory and empirical studies.

### Theoretical Implications

In general, our findings regarding all four hypotheses support the process model of retirement (e.g., Wang & Shultz, 2010) in its assumption that job and organizational factors might act as antecedents of retirement decisions. Our findings contribute to the model first by adding psychological empowerment as a concrete antecedent at the job and organization level. Second, as psychological empowerment was related not only to retirement timing but also to the bridge employment decision making, our findings suggest that the same job and organizational factors experienced during working life can influence retirement decisions across phases and have a lasting effect on the retirees’ lives.

The results related to Hypothesis 1 are in line with previous findings showing that person-job variables are important factors for the DRA and the ARA. For example, research indicates that job control is associated with later retirement intentions and later actual retirement (see a review Browne et al., 2018). Going beyond the existing literature, our findings contribute to the literature in three unique ways. First, they extend findings from Schermuly et al. (2017), who found that psychologically empowered employees desired to retire later. By including ARA, we respond to calls for more research on retirement outcomes that goes beyond intentions (see Browne et al., 2018). Second, we transferred psychological empowerment to the retirement context, suggesting that retirement age, along with constructs such as turnover intention and organizational commitment, is another important outcome of psychological empowerment in the organizational context. Third, our results support the theory of planned behavior (Ajzen, 1991) as well as its theoretical extension that proposes intentions (in our case, DRA) as a mediating variable (see Perugini & Bagozzi, 2001). DRA and ARA were highly correlated in our sample (*r* = 0.67), which implies that the desire to retire at a certain time is realizable. This might be due to the German retirement system, which allows for flexible timing. In Sweden, where there is also no fixed retirement age, panel studies have found similarly strong relationships between DRA and ARA (Örestig et al., 2013; Solem et al., 2016). In the present study, however, DRA was slightly higher than ARA, indicating that the participants retired earlier than they desired. Solem et al. (2016) identified conditions, such as poor health and education, that increased the risk of retiring earlier than preferred. More research is needed to further investigate boundary conditions of the relationship between DRA and ARA, as well as the psychological consequences of not retiring at one’s DRA.

Additional analyses of the role of the individual empowerment facets revealed that only the competence facet was significantly related to the DRA and the ARA, resulting in full mediation by the DRA. This finding is interesting and might be in line with research on employability, which can be defined as “the continuous fulfilling, acquiring or creating of work through the optimal use of competences” (Heijde & Van Der Heijden, 2006, p. 453). Researchers have argued that employability is an important factor in the motivation to continue working (see, e.g., Pak et al., 2019).

The results regarding Hypothesis 2a are in line with previous findings on antecedents of bridge employment at the person-job level. For example, a study by Fasbender et al. (2016) found that the social meaning of work (e.g., having contact with others) and the personal meaning of work (e.g., having a meaningful task) were positively related to the likelihood of engaging in bridge employment. Our results differ from those of Fasbender et al. (2016) in that they do not focus on employees’ beliefs about work in general, but on employees’ cognitions that are shaped by their immediate work environment. From a theoretical perspective, our results support continuity theory (Atchley, 1989), as individuals who were psychologically empowered *before* retirement showed more engagement in paid work *after* retirement (i.e., engagement in bridge employment). This could imply that psychologically empowered individuals are likely to seek out contexts in which they can continue to experience psychological empowerment. This finding is consistent with empowerment theory, according to which psychologically empowered employees are more likely to take an active orientation toward work and go the “extra mile” Spreitzer (2008). Thus, it seems plausible that they are less likely to completely withdraw from work compared to less empowered older individuals. In light of continuity theory, one may conclude that psychological empowerment during working life—or its continued experience after retirement—might help older individuals to maintain stability during potentially disruptive life changes such as retirement. Future research could test this assumption by investigating the relationship between psychological empowerment during working life and post-retirement outcomes such as retirement satisfaction, psychological health, and/or physical health.

In Hypothesis 2b, we compared paid and unpaid forms of later life work. Our results provide indications that psychological empowerment is more strongly associated with bridge employment than with volunteering. Results regarding Hypothesis 2b might also support continuity theory (Atchley, 1989), according to which middle-aged and older individuals aim to preserve structures by “applying familiar strategies in familiar arenas of life” (p. 183)—context and familiarity have likely played a role in maintaining continuity in the present study. Furthermore, the fact that bridge employment and volunteering were not significantly correlated might speak against the so-called trade-off hypothesis (see Vangen et al., 2021): Engaging in bridge employment did not decrease the likelihood of being engaged in volunteering and vice versa. Thus, it seems that the two activities did not compete for the retirees’ resources in our study. Vangen et al. (2021) did find a negative relationship between paid work and volunteering in a sample of Norwegian seniors aged 62–75 over a 10-year period. However, the negative relationship was found only in participants with full-time employment (not among those with part-time employment), and the relationship did not apply for participants with former experience in voluntary work. The authors conclude that, in order to increase older people’s engagement in both paid and unpaid work, it might be beneficial to stimulate combinations of both forms in late careers (Vangen et al., 2021). Thus, while psychological empowerment at the workplace could be used to stimulate paid work after retirement (i.e., bridge employment), informing older individuals early about possibilities for volunteering could help to engage them in volunteer work before they retire (e.g., Hansen & Slagsvold, 2020).

Additional analyses regarding Hypotheses 2a and 2b revealed that none of the facets of the empowerment construct individually predicted bridge employment. This might indicate that the relationship between psychological empowerment and bridge employment is driven by the “Gestalt” of psychological empowerment (i.e., the joint presence of all facets; see Spreitzer, 1995). However, it might also have resulted from methodological limitations. We will discuss this in the Limitations section.

Finally, our results regarding Hypothesis 2c indicate that the level of physical limitations moderates the relationship between psychological empowerment and bridge employment decision making. This finding is in line with previous research on constraining variables. For example, Zhan et al. (2013) identified economic stress as a boundary condition for the relationship between career commitment and career-based bridge employment decision making and the relationship between affective commitment to organization and organization-based bridge employment decision making, such that high economic stress weakened both relationships. Both these findings and our finding support the model by Beehr and Bennett (2015), which stresses the role of situational constraints in the bridge employment decision-making process. Future research could combine our study with the study by Zhan et al. (2013) by investigating whether empowered employees are more likely to take bridge employment at the same organization and identify constraining variables that could be at play in this relationship.

### Practical Implications

One practical implication of the strong relationship between DRA and ARA could be for policymakers to assess DRA among older employees to predict changes in the public pension system. Likewise, organizations might consider assessing their older employees’ DRAs for more accurate staff planning. Furthermore, organizations wanting to retain older employees—whether through delayed retirement or bridge employment—could consider enhancing psychological empowerment cognitions in their employees. They might apply general approaches that have been identified as contextual antecedents of psychological empowerment, for example, high-performance managerial practices and sociopolitical support (see meta-analyses by Llorente-Alonso et al., 2023 and Seibert et al., 2011). More specific human resource management measures for older employees should also be considered. For example, measures against age discrimination seem important, as research indicates negative associations between age discrimination and psychological empowerment, which in turn leads to a lower DRA (Schermuly et al., 2014). Optimally, specific measures for empowering older employees should be developed. One practical suggestion for policymakers thus might be to consider funding research in this interdisciplinary research field, as knowledge in this area could help motivate older workers to remain active longer and thereby reduce the financial burden on public pension and health care systems in aging societies. It would be especially important to encourage research on psychological empowerment that investigates workers older than 50 years, as a recent meta-analysis by Llorente-Alonso et al. (2023) indicates that few empirical studies have included this population. Finally, organizations that want to retain older employees after retirement should start promoting the physical health of their employees early because, according to our findings, the relationship between psychological empowerment and bridge employment disappears when employees report high levels of physical limitations. Research informs us that one way leaders can promote the subjective health of their subordinates is to practice respectful leadership, which in turn might even increase older workers’ DRA (see Wöhrmann et al., 2017).

### Limitations

The limitations of the present study result mainly from the archival nature of the panel data. First, as psychological empowerment and DRA were collected at the same measurement point, common method bias may have altered their relationship (Podsakoff et al., 2012). At least the criterion in this mediation model, ARA, was measured with a considerable time lag (i.e., three years) and presented a more objective measure. However, we still cannot draw causal conclusions about the relationship between psychological empowerment and engagement in later life work. Although we had two measurement points, the focal variables were not measured at both time points. Future researchers should conduct longitudinal or preferably interventional studies to substantiate our assumption that psychological empowerment increases the likelihood of engaging in later life work and not (exclusively) vice versa. Another potential limitation is that most of the constructs were measured by single items or reduced scales due to the telephone interview method. For example, the empowerment construct was limited to four items. This factor plus the constructs’ multidimensionality led to a low omega coefficient. Future research could replicate our findings with the standard instrument by Spreitzer (1995) using an online questionnaire. Furthermore, future research could also test the measurement invariance of the empowerment scale related to age, in particular when investigating more age-diverse samples (for a brief tutorial, see Guo et al., 2023). Another potential limitation, which could be at least partly due to measurement problems, is that the effect sizes seemed relatively small. However, recent research suggests that effect sizes that seem small when judged by conventional standards (e.g., by Cohen’s guidelines) may not be weak at all when more domain-specific standards are applied (e.g., by evaluating correlations from multiple relevant meta-analyses and defining cut-offs based on the 25th, 50th, and 75th percentiles of reported effect sizes; see Davenport et al., 2022). Moreover, even minor individual differences in the timing of retirement and/or the choice of bridge employment are likely to have drastic effects on social security systems through cumulative effects, given how many older workers are likely to retire in the coming years. The study sample itself is another methodological limitation. Participants were mostly highly educated white-collar workers. This might have inflated the correlation between DRA and ARA, as research indicates that a lower education level increases the probability of retiring before one’s DRA (Solem et al., 2016). Furthermore, our participants were employed individuals aged between 54 and 65 years at the first measurement. Thus, we investigated a highly selective sample of the population, excluding individuals who were unemployed at that time or might have already retired involuntarily. Future research should therefore also examine workers aged 50 or younger to explore involuntary retirement and ways to prevent it. Another limitation is that the findings can be extrapolated to other countries only to a limited extent due to differing retirement and work regulations. Finally, it is important for future research to examine the incremental validity of psychological empowerment for predicting retirement timing and engagement in later life work against alternative, conceptually meaningful factors such as psychological capital, person–environment fit, or job involvement.

## Conclusion

This study aimed to investigate the relationship between psychological empowerment before retirement and later life work. The three most important findings of this study are as follows: (1) Older workers with higher levels of psychological empowerment not only wanted to retire later but indeed retired later, (2) older workers with higher levels of psychological empowerment were more likely to engage in bridge employment, and (3) the positive relationship between psychological empowerment and bridge employment disappeared when older workers experienced high levels of physical limitations. These findings suggest that psychological empowerment might help to retain older workers in the workforce. At the same time, our study points to the limitations of psychological empowerment, as the latter became insignificant in predicting bridge employment when high levels of physical limitations were reported. Thus, we hope that our study sensitizes both researchers and practitioners for the importance of a holistic approach when studying or promoting longer working lives. Lastly, we encourage future researchers to replicate our findings outside the interview context.
